# No Association between Mycotoxin Exposure and Autism: A Pilot Case-Control Study in School-Aged Children

**DOI:** 10.3390/toxins8070224

**Published:** 2016-07-20

**Authors:** Jennifer Duringer, Eric Fombonne, Morrie Craig

**Affiliations:** 1 Department of Environmental & Molecular Toxicology, Oregon State University, 139 Oak Creek Building, Corvallis, OR 97331, USA; jennifer.duringer@oregonstate.edu; 2 Department of Psychiatry, Institute for Development & Disability, Oregon Health & Science University, 840 SW Gaines St., Portland, OR 97239, USA; 3 College of Veterinary Medicine, Oregon State University, 105 Magruder Hall, Corvallis, OR 97331, USA; a.morrie.craig@oregonstate.edu

**Keywords:** autism, mycotoxins, environmental, urine

## Abstract

Evaluation of environmental risk factors in the development of autism spectrum disorder (ASD) is needed for a more complete understanding of disease etiology and best approaches for prevention, diagnosis, and treatment. A pilot experiment in 54 children (*n* = 25 ASD, *n* = 29 controls; aged 12.4 ± 3.9 years) screened for 87 urinary mycotoxins via liquid chromatography-tandem mass spectrometry to assess current exposure. Zearalenone, zearalenone-4-glucoside, 3-acetyldeoxynivalenol, and altenuene were detected in 9/54 (20%) samples, most near the limit of detection. No mycotoxin/group of mycotoxins was associated with ASD-diagnosed children. To identify potential correlates of mycotoxin presence in urine, we further compared the nine subjects where a urinary mycotoxin was confirmed to the remaining 45 participants and found no difference based on the presence or absence of mycotoxin for age (*t*-test; *p* = 0.322), gender (Fisher’s exact test; *p* = 0.456), exposure or not to selective serotonin reuptake inhibitors (Fisher’s exact test; *p* = 0.367), or to other medications (Fisher’s exact test; *p* = 1.00). While no positive association was found, more sophisticated sample preparation techniques and instrumentation, coupled with selectivity for a smaller group of mycotoxins, could improve sensitivity and detection. Further, broadening sampling to in utero (mothers) and newborn-toddler years would cover additional exposure windows.

## 1. Introduction

Autism Spectrum Disorder (ASD) or Pervasive Developmental Disorders (PDD; from this point forward we elected to use ASD for simplification) are lifelong conditions characterized by pervasive impairments in social reciprocity and communication, stereotyped behaviors, and restricted interests [1,2]. Recent reviews have concluded that current best estimates for the prevalence of ASD lie between 0.7% and 1% [3,4,5], although some studies have yielded higher estimates [6,7,8]. The most recent prevalence figure for the United States is 1.47%, determined by the Autism and Developmental Disabilities Monitoring program supported by the Centers for Disease Control and Prevention [9]. Although there is no doubt that the prevalence of ASD has increased over the last 30 years [4], interpretation of this trend remains uncertain. Variability in survey methods, changes in diagnostic concepts, and broadening of definitions, diagnostic substitution, and improved identification and awareness have all contributed [4,5]. However, the hypothesis that an increase in the underlying incidence of ASD also accounts for some of the upward trend in prevalence cannot be ruled out. If confirmed, it could point to environmental risk factors as playing an etiological role in ASD alongside that of well-established genetic factors [10,11]. Consequently, examining environmental factors that may contribute to an increase in ASD is a priority of the autism research agenda.

Mycotoxins are “natural” environmental contaminants, defined as secondary metabolites produced by fungi that reside in our food supply and on every surface in our environment. Precipitation and climatic conditions conducive to the growth of molds result in production of mycotoxins—both in residences [12,13,14,15,16] and in cereals, grains, and other susceptible crops as harvested or in food storage facilities [17,18]. *Aspergillus*, *Claviceps*, *Fusarium,* and *Penicillium* are some of the main fungal genera that produce these compounds. Exposure is generally through the diet (ingestion), although inhalation or skin contact with spore-borne toxins are also important routes of introduction to the host [19].

Neurological and developmental effects from mycotoxin exposure have been reported in both humans and animals [17,19,20,21,22]. A review on fumonisins as a common contaminant of maize suggested that they are risk factors for neural tube, craniofacial, and other birth defects arising from neural crest cells because of their interference with folate utilization [23]. Another study in mothers who ingested moderate-significant quantities of tortillas during gestation along the Texas-Mexico border found fumonisin-contaminated corn tortillas to be linked to increased neural tube defects and fetal death; women in the highest quartile were estimated to have consumed 650–9441 ng/kg body weight of fumonisins [24]. Ochratoxin A has been shown to induce teratogenic effects in neonates (rats and mice) exposed in utero, characterized by microcephaly and modification of brain levels of free amino acids [25,26]. Prenatal exposure to 1.2 mg/kg body weight over four days of aflatoxin B1 produced a delay of early response development, impaired locomotor coordination, and impaired learning ability in the offspring of rats exposed to this mycotoxin during the middle of gestation [27]. T-2 and HT-2 toxin showed cytotoxic activity on the blood-brain barrier in vitro, with indications that these compounds are able to enter the brain [28]. Further, aflatoxin B1, B2, and G1 were detected in the serum (3.5 µg/L), urine (0.3–18.8 µg/L), and amniotic fluid (4.3 µg/L) of pregnant women [29]; deoxynivalenol (DON) was transported in an ex vivo placental model [30] and detected in the urine of pregnant women from Croatia in their third trimester (18.3 µg/L in addition to DON conjugates) [31]; and zearalenone and its metabolites were detected in fetuses of rats administered this compound two times during pregnancy (1.1–65.3 ng/g) [32], confirming that mycotoxins are present and transmissible in fetal-maternal biological fluids.

Whether a connection exists between mycotoxins in the environment and the development of ASD has not been directly investigated. Two studies that examined ASD risk in relation to wet weather conditions could be regarded as proxy measures for mycotoxin levels, although this type of inference is highly speculative at this stage. In one, severity of exposure to tropical storms and hurricanes pre-natally was positively associated with autism prevalence from storm events in Louisiana from 1980 to 1995, especially in mothers who were in mid- or late gestation [33]. In the second, county precipitation levels were positively correlated with rates of ASD in schools from counties of three western states in the USA, although investigation relied on a weak ecologic epidemiological design, likely making the results confounded (Waldman, et al., 2008) [34]. Finally, a small preliminary study suggested that individual exposure to mold increased the severity of neurophysiological abnormalities seen in autistic children [35]. The authors compared six autistic children exposed to molds and mycotoxins in the home to eight autistic children with no mycotoxin exposure and 29 non-autistic children, and found that the mycotoxin-exposed autistic group had a 1.8-fold higher number of neurobehavioral abnormalities versus the non-mycotoxin autistic group, and a 12.2-fold higher number of abnormalities than the non-autistic children. The methods used to determine mycotoxin exposure utilized either culturing of mold or air from the patients’ homes or antibody detection of the sera to selected mycotoxins [36], but no quantifiable results were reported. To our knowledge, no other survey of mycotoxin exposure and determination of associative influence of these compounds on development of ASD has been conducted. Thus, we performed a pilot study where we recruited children with ASD and age-matched controls in order to survey their current exposure to a range of mycotoxins using liquid chromatography-tandem mass spectrometry (LC-MS/MS).

## 2. Results

Table 1 provides descriptive data of our sample population. The overall mean age of the 54 participants was 12.4 years (SD = 3.9; range: 5 to 20), with no statistical difference between the groups. As expected, boys were overrepresented in the ASD case group with a male:female ratio of 5.4:1. Similarly, the vast majority of cases had an Individualized Educational Plan (IEP) as compared to a fourth of the controls. In this predominantly clinical sample, there was no difference for selective serotonin reuptake inhibitors (SSRI) or other medication usage. Participants attended all school grades from pre-K (*n* = 5) to grade 11 (*n* = 3), with no difference across groups. Participants resided in six counties, with over 75% living in the two most populous counties (Multnomah and Washington) of the greater Portland agglomeration. The proportion of households with pets was comparable in the two groups. Characteristics of the ASD cases were typical of a school-age sample, with a relatively high proportion of high-functioning subjects. Thus, 60% of the children had fluent language as compared to 16% with no language or only single word combinations. Of 17 children with information, six (35.3%) had either a Vineland composite score or an IQ score below 70.

Results from the urinary mycotoxin screen are given in Table 2. The mycotoxins that were detected were zearalenone (ZEN), zearalenone-4-glucoside, 3-acetyldeoxynivalenol, and altenuene. These were detected near the lower limit of quantitation for all except 3-acetyldeoxynivalenol. Each compound was also generally evenly distributed between both the ASD and control groups. In addition, the qualifier transition for deoxynivalenol was strong in six samples (five control and one ASD); however, the quantifier transition (found at a ratio of 1:4.72 to the qualifying transition [37]) was either at or below the noise level, making confirmation of this mycotoxin negative. (The limit of quantitation, LOQ, for deoxynivalenol was 50 µg/L). All mycotoxins detected were from separate individuals in both groups, giving 9/54 (17%) of the participants being positive for at least one mycotoxin.

In order to identify potential correlates of mycotoxin presence in urine, we further compared the nine subjects where a urinary mycotoxin was confirmed to the remaining 45 participants with no mycotoxins, pooling cases and controls together. No difference was found according to the presence or absence of mycotoxin for age (*t*-test; *p* = 0.322), gender (Fisher’s exact test; *p* = 0.456), exposure or not to SSRIs (Fisher’s exact test; *p* = 0.367), or to other medications (Fisher’s exact test; *p* = 1.00).

## 3. Discussion

Increasing attention has been given to fungal infections and their effect on human health, particularly in individuals that are immunocompromised [38,39,40]. Whether the pathology exhibited in the human host from penetration and propagation of the fungal organism in mycoses is exacerbated by the production of mycotoxins is an understudied concept. In addition, one fungal species may produce several chemically distinct mycotoxins, each generating unique physiological sequelae, and/or many fungal species may colonize food/feed or air that is ingested which further complicates a thorough comprehension of the toxicological response elicited in the animal host [17].

Denning, et al. (2015) [40] cite development of faster diagnostics as a major barrier to better understanding and detecting fungal diseases. Indeed, monitoring of susceptible crops [18] and biomatrices of vulnerable individuals is crucial to preventing the development of mycotoxicoses. Analytical instrumentation technologies have only recently allowed limits of detection to be lowered such that robust, quantitative assessments of mycotoxin exposure at biologically relevant concentrations are now possible, primarily using non-invasive urinary specimens [31,41,42,43]. This direct biomonitoring approach, which often includes relevant conjugates of mycotoxin targets—thereby eliminating the need for enzymatic hydrolysis and complicated sample prep—is gaining preference to the traditionally used indirect alternative of examining mycotoxin contamination in food and pairing that with food consumption in the population studied to estimate exposure. However, new conjugates, metabolites and other “masked” mycotoxin products are continually being discovered, making the task of complete mycotoxin exposure assessment ever-changing [18,44].

In the current study, we utilized mass spectrometry to conduct the first (to our knowledge) assessment of mycotoxin exposure in patients diagnosed with ASD. We detected four individual mycotoxins in 9/54 (17%) of the samples—specifically zearalenone, zearalenone-4-glucoside, 3-acetyldeoxynivalenol, and altenuene. Each was approximately evenly distributed between both groups; thus, no one mycotoxin or group of mycotoxins appears to be associated with the environment of the ASD children we assessed. Age, gender, presence/absence of SSRIs, or if the child was taking other medications was compared in the nine individuals who had mycotoxin detects against the 45 who did not; none of these variables were deemed significant.

Of the compounds detected, zearalenone and its metabolites (zearalenone-4-glucoside in the current study) and the trichothecenes (3-acetyldeoxynivalenol) are frequently targeted and identified in human urinary mycotoxin biomarker surveys across the globe. For example, a study on 120 individuals in Nigeria identified some of these (mean 13.4 µg/L for total DON + ZEN) and others (0.2–4.6 µg/L) for a total of eight compounds in children, adolescents, and adults, with those from rural areas more affected than those in cities [45]. Another survey of 220 Cameroonian children aged 1.5–4.5 years old detected zearalenones, deoxynivalenol, fumonisin B1, aflatoxin M1, and ochratoxin A (range 0.18–3.94 µg/L) in 73% of the urine samples, and found higher loads in breastfed babies versus those who were fully weaned [46]. Urine samples from a broad spectrum of ages in one of the first large-scale studies on mycotoxin exposure in Belgium (155 children and 239 adults) confirmed nine out of 33 urinary mycotoxin biomarkers examined, including members from the zearalenone and trichothecene groups (range 0.03–91.7 µg/L) [47]. The authors concluded that young children should receive special consideration in risk assessment calculations, as their relatively higher food intake per kg body weight resulted in a larger percentage of them exceeding tolerable daily intakes. In fact, this is a common finding in mycotoxin exposure assessment studies, which emphasize caution and concern for children and their increased susceptibility to the adverse health effects of mycotoxins, especially when compounded by other factors like poor nutrition and chronic infections, which affect proper growth and development [46,48,49,50,51]. Surveys such as these are just beginning to directly estimate global mycotoxin exposure. As many authors assert, large-scale, multi-location studies straddling all age groups that include multi-time point sampling from the same individuals are needed to establish both periodic and long-term levels of mycotoxin load, to be followed by studies which correlate exposure to toxicity and disease development.

While we utilized the best available detection methodology for this study, a number of technical approaches could be used to improve sensitivity and therefore detection of mycotoxins, including: employing more sophisticated sample preparation techniques such as solid phase extraction or immunoaffinity columns; being more discriminating in the compounds chosen for measurement and the time spent scanning by the instrument—e.g., selecting 15 mycotoxins and their metabolites as in Warth, et al. (2012) [43] instead of scanning for 87 and/or the implementation of scheduled multiple reaction monitoring (MRM) transitions; and use of a more advanced triple quadrupole or time-of-flight mass spectrometer capable of achieving lower detection limits. This may help confirm and quantify the presence of commonly-detected mycotoxins such as DON, which had trace peak intensities in six samples, and a number of samples which had the qualitative but not the quantitative transition present, such as 3-acetyl-DON and DON-3-glucoside. Agricultural products, and consequently, urine from people of Western nations are frequently contaminated with DON [47,52,53], so finding it and its glucuronide conjugates would be expected. Lastly, the caveat of the “dilute and shoot” method used here is that, while it has speed and cost advantages, along with full recovery of a chemically diverse array of compounds and their conjugates, it was optimized to capture high-to-moderate exposures rather than low or background levels [54]. With these analytical limitations in mind, this survey showed that there was no overwhelming load of urinary mycotoxin biomarkers present in the population studied.

In addition, specimens were obtained from children with an age of 12.4 ± 3.9 years, well after the in utero or infant-toddler exposure window when ASD develops and is diagnosed. Urinary sampling across multiple time points in the mother and children in the earlier years would provide a more thorough evaluation of mycotoxin exposure and possible association with ASD incidence. Further, it is unknown if the children used in this study were in the same residence as when the mother was pregnant and the child was in its first few years of life. Even if that were the case, mycotoxin load in an individual can fluctuate based on seasonal and other factors affecting fungal growth in the environment, in addition to variances through dietary exposure. As most mycotoxins excreted by the kidney into the urine are cleared within 48 h [47], a study such as the one presented here gives only a recent snapshot of mycotoxin intake. Lastly, other considerations, such as altered immune regulation [55] and gastrointestinal microbiota profiles [56] in autistic individuals may play a role in mycotoxin metabolism and therefore modify ultimate exposure of mycotoxins in susceptible children, which should be integrated into the larger research picture in future studies.

## 4. Conclusions

In conclusion, our preliminary survey study found no association with increased urinary mycotoxin biomarkers and ASD. More sophisticated sample preparation techniques and instrumentation, coupled with selectivity for a smaller group of mycotoxins, could improve sensitivity and detection. In addition, samples from in utero (mothers) and newborn-toddler years would cover additional exposure windows to better qualify if mycotoxin load is correlated with ASD incidence.

## 5. Materials and Methods

### 5.1. Sample Selection

Children with ASD (*n* = 25) participating in the Autism Clinical Program at the Child Development and Rehabilitation Center (CDRC), Oregon Health & Science University (OHSU), and their parents were approached for participation. Diagnosis was verified using DSM-IV criteria and confirmed by clinicians familiar with the children. Controls (*n* = 29) were selected from general pediatric clinics or from community friends of index families. Participants were group-matched on age. A brief questionnaire was completed by the parents, including basic socio-demographic information, county of residence (an administrative area that can be linked to an annual precipitation level), school grade, whether or not the child had an IEP at school, whether or not the child was taking medication (SSRI or any other prescribed drug), and whether or not a pet was present in the household. For children with ASD, additional questions were asked about language proficiency (fluent; phrase speech; single words combinations; nonverbal) and the presence of a relative with ASD; when available, we extracted data on IQ and Vineland scores assessing cognitive level and adaptive behavior from medical records.

### 5.2. Specimen Collection

All participants were provided with a four ounce sterile cup with screw-top lid and instructed on proper sample collection. Urine from the first morning micturition was collected at home by the subject, assisted by a parent as needed. Urines were promptly transported by participants to OHSU and immediately frozen. Frozen specimens were subsequently transported to Oregon State University (OSU) where they were stored at −20 °C until mycotoxin analysis.

### 5.3. Chemical Analysis

Urine was prepared for mycotoxin analysis using a dilute and shoot approach [43]. Urine was removed from the freezer and allowed to reach room temperature, then centrifuged for 3 min at 3331× *g*. Supernatant (100 µL) was removed and added to 900 µL of 10:90 acetonitrile:water (acetonitrile was LC-MS grade and purchased from Millipore (Billerica, MA, USA); 18 mΩ water was obtained from an ELGA Ultra PureLab water system (Cary, NC, USA). The mixture was vortexed then sealed for analysis by LC-MS/MS. 

Mycotoxins were detected on an AB/SCIEX 3200 QTRAP LC-MS/MS system (Applied Biosystems, Foster City, CA, USA) via electrospray ionization, with separation performed using a Perkin Elmer (Waltham, MA, USA) Series 200 HPLC connected to a Gemini C18 column (150 × 4.6 mm, 5 µ, Phenomenex (Torrance, CA, USA)) with a 4 × 3 mm security guard cartridge of similar packing [37]. Mobile phases consisted of 5 mM ammonium acetate and methanol:water:acetic acid in a ratio of 10:89:1(*v*/*v*/*v*) (A) or 97:2:1 (B) and were run in a gradient program at 1 mL/min. (Ammonium acetate (HPLC grade) and acetic acid (LC-MS grade) were purchased from Sigma Aldrich (St. Louis, MO, USA); methanol was LC-MS grade and was purchased from Millipore). Each sample was run in triplicate; each replicate had two injections taken, one each for both positive and negative modes, which captured 87 mycotoxins via MRM of two transitions (quantitative and qualitative) per compound. Mass spectrometer settings were as described in Sulyok, et al., (2007) [37]. Quantitative analyses were conducted blind to the group status of each child specimen using Analyst, MultiQuant (Applied Biosystems), and Excel (Microsoft, Redmond, WA, USA). The presence of a mycotoxin was confirmed when the signal was equal to or greater than a signal to noise (S/N) ratio of 3:1 (limit of detection, LOD), and both quantitative and qualitative transitions were present. Standards were purchased for detected mycotoxins to generate standard curves in neat solvent for quantitation (zearalenone and 3-acetyldeoxynivalenol from Romer Labs, Getzersdorf, Austria). The limit of quantitation (LOQ) was defined as the concentration at which the analyte had a precision of 20% and accuracy of 80%–120% [57], which was 50 µg/L for both compounds. As there was no commercially available standard for zearlenone-4-glucoside, its peak intensity was compared to its parent zearalenone for relative quantitation. In addition, altenuene had no standard available for purchase, so its quantity is described relative to the peak intensity defined for the LOD.

### 5.4. Ethical Approval

The study was conducted in accordance with the rules of the Declaration of Helsinki of 1975, revised in 2008. Written consent was obtained from participants. Approval to conduct the study was obtained from the OHSU Institutional Review Board on 30 April 2013 (IRB 9424). Vouchers with a $20 value were provided to participants upon receipt of the urine sample.

### 5.5. Statistical Analyses

Conventional statistics were used to compare characteristics of cases and controls (*t*-test and Fisher’s exact tests). Analyses were performed in SPSS 22.1 (IBM, Armonk, NY, USA) and in GraphPad Prism (GraphPad Software, La Jolla, CA, USA). Throughout, a *p*-Value < 0.05 was retained to denote statistical significance.

## Figures and Tables

**Table 1:** Sample characteristics.

Variable	Controls (*n* = 29)	Cases (*n* = 25)	*p*
Age: mean (SD)	12.8 (4.0)	11.8 (3.8)	NS ^3^
Male: *n* (%)	12 (41.4)	21 (84.0)	0.001
Has an IEP ^1^: *n* (%)	8 (27.6)	23 (92.0)	<0.001
Takes an SSRI ^2^: *n* (%)	5 (17.2)	6 (24.0)	NS
Takes other medications: *n* (%)	15 (51.7)	13 (52.0)	NS
Has pets at home: *n* (%)	23 (79.3)	16 (64.0)	0.21

^1^ Individualized Educational Plan; ^2^ Selective Serotonin Reuptake Inhibitor; ^3^ NS: Not significant.

**Table 2:** Occurrence of urinary mycotoxins from children with autism spectrum disorder and controls.

Mycotoxin	Autism Spectrum Disorder (*n* = 25)		Typically Developing (*n* = 29)	
	Number (%) of Positive Samples	Relative Conc (µg/L) ^a^	Number (%) of Positive Samples	Relative Conc (µg/L) ^a^
Zearalenone	1 (4%)	<LOQ	0 (0%)	-
Zearalenone-4-glucoside	1 (4%)	<LOQ ^b^	1 (3%)	<LOQ ^b^
3-Acetyldeoxynivalenol	0 (0%)	-	1 (3%)	3.1
Altenuene	3 (12%)	LOD ^c^	2 (7%)	LOD ^c^
Any mycotoxin	5 (20%)	-	4 (14%)	-

^a^ Numerical values represent an average for the samples in that group. <LOQ = sample detected below the limit of quantitation. LOQ for zearalenone and 3-acetyldeoxynivalenol was 50 µg/L; ^b^ No standard was commercially available so peak intensity was compared to the parent compound; ^c^ No standard was commercially available so quantities are described relative to the peak intensity defined for the limit of detection (LOD).
